# Barriers to early presentation and diagnosis of breast cancer among African women living in sub-Saharan Africa

**DOI:** 10.1371/journal.pone.0171024

**Published:** 2017-02-13

**Authors:** Cynthia Pomaa Akuoko, Ernestina Armah, Theresa Sarpong, Dan Yedu Quansah, Isaac Amankwaa, Daniel Boateng

**Affiliations:** 1 Christian Service University College, Kumasi, Ghana; 2 Graduate School of Public health, Seoul National University, Seoul, South Korea; 3 Garden City University College, Kumasi, Ghana; 4 Julius Global Health, University Medical Center, Utrecht University, the Netherlands; 5 School of Public Health, Kwame Nkrumah University of Science and Technology, Kumasi, Ghana; Universita degli Studi di Torino, ITALY

## Abstract

**Background:**

Breast cancer (BC) has been described as the leading cause of cancer deaths among women especially in the developing world including sub Saharan Africa (SSA). Delayed presentation and late diagnosis at health facilities are parts of the contributing factors of high BC mortality in Africa. This review aimed to appraise the contributing factors to delayed breast cancer presentation and diagnosis among SSA women.

**Methods:**

Five databases encompassing medical and social sciences were systematically searched using predefined search terms linked with breast cancer presentation and diagnosis and sub Saharan Africa. Reference lists of relevant papers were also hand searched. Quality of quantitative and qualitative articles were assessed using the National Institute of Health (NIH) Quality Assessment Tool for Observational Cohort and Cross-Sectional Studies and the Critical Appraisal Skills Programme (CASP) quality appraisal checklist. Thematic analysis was used to synthesize the qualitative studies to integrate findings.

**Results:**

Fourteen (14) quantitative studies, two (2) qualitative studies and one (1) mixed method study merited inclusion for analysis. This review identified low knowledge of breast cancer among SSA women. This review also found lack of awareness of early detection treatment, poor perception of BC, socio-cultural factors such as belief, traditions and fear as factors impacting African women’s health seeking behavior in relation to breast cancer.

**Conclusion:**

Improving African women’s knowledge and understanding will improve behaviors related to breast cancer and facilitate early presentation and detection and enhance proper management and treatment of breast cancer.

## Introduction

Incidence and mortality rates for cancer has increased over the second half of the 20th century and are likely to continue to surge substantially according to World Health Organization (WHO) projections [1]. In 2012, the world health cancer report estimated an unprecedented 14 million new cases and 8.2 million cancer related deaths, with the figure expected to rise by almost 70% over the next 2 decades [2]. Breast cancer is now the most common cancer both in developed and developing regions with around 690,000 new cases being diagnosed annually in the developed regions and around 92,000 in Africa [3]. BC is the leading cause of cancer death in females’ worldwide [4]. It accounted for 14% of the total cancer deaths in 2008. Although BC is the leading cause of cancer deaths in females worldwide, the fatality rates tend to be higher in economically developing countries. The incidence of breast cancer in Ghana is estimated to be 25 cases per 100,000 population compared to 93 per 100,000 in the USA. However, mortality is 12 per 100,000 in Ghana compared to 15 per 100,000 in the USA [5]. There is evidence of emerging disparity in long-term mortality trends, with mortality rising in parallel with incidence in some countries yet declining in others despite rising incidence rates [6]. In developed countries, although incidence rates are high for BC, death rates have been decreasing over the past 25years [7]. A lot of factors might account for this growing disparity between the economically developed and developing countries.

Variation in incidence rates may largely stem from greater availability of early detection measures as well as health seeking behaviors of women in developed countries [6]. Moreover, effective therapy may help lower BC death rates after detection [8]. Patient’s delay (delay between individuals’ first awareness of breast abnormality and initial medical consultation) is a common factor that contributes to late detection of BC and presentation at any healthcare facility. It is believed that around 20–30% of women with symptoms of BC wait three months before consulting their physicians [9].

Studies undertaken in Africa suggest low knowledge of breast and cervical cancer awareness [10–12]. This tends to impact on attitudes to uptake of screening resulting in late diagnosis in many women. Again, fatalism, fear, embarrassment, lack of trust in health services, lack of education has been cited as barriers to early presentation of the disease in African American women [13–16]. These conditions affect not only the health and lives of the women, but also their children, families, communities and the nation at large. Research stipulates that, early detection is an important determinant for better prognosis of several kinds of cancer including BC [17]. Furthermore, treatment of early stage BC is typically simpler and more cost effective than treatment of its advanced episodes [18]. Where healthcare resources are scarce, early detection positively impacts the delivery of BC treatment, that is, treatment in an earlier stage is likely to be less complex and more affordable [19].

The disparity in BC outcomes in women living in developing and developed countries provides the momentum for this paper to review research on barriers to early presentation and diagnosis of BC in women from Africa. This review is also relevant as barriers may not be limited to early presentation for BC alone. Findings from this review can potentially aid in drafting measures to improve the knowledge and health seeking behaviors of African women. This review was conducted with the aim to synthesize research evidences on the barriers to early presentation and diagnosis of breast cancer among women in sub-Saharan Africa.

## Materials and methods

### Search strategy

A comprehensive search strategy was developed to find published studies on BC knowledge and health seeking behaviors of women in sub Saharan Africa. The sub Saharan region was classified based on the United Nations classification of countries [20]. The search strategy (Appendix 1) was run in Medline then later adapted for, and run across selected relevant databases. The databases searched were: CINAHL (1990 to December 2015), Medline (1990 to December 2015), Cochrane (1990 to December 2015), PsychInfo (1990 to December 2015), Embase (1990 to December 2015). Reference list of studies included in the review were hand searched along with titles and abstracts of African medical journals such as the South African medical journal, West African medical journal, East African medical journal and Central medical journal. All studies identified during the search were assessed for relevance to the review based on the information provided in the title and abstract. For all papers that appeared to meet the inclusion criteria, a full report was retrieved. This was again assessed for applicability against the inclusion criteria in order to determine relevance to the review objective.

### Inclusion criteria

Studies that were included in this review were of any design (qualitative and quantitative) published in English language peer-reviewed journals, and primary research articles, that identified barriers to early presentation and diagnosis of BC in women of African descent living in Africa. This included studies that explored knowledge of BC, studies of attitudes or barriers to breast screening, studies that explored attitude to and undertaking of breast self-examination (BSE). Studies that explored health seeking behaviors regarding BC, factors affecting women’s return for follow-up following abnormal test results, diagnostic delays due to service-related factors after suspicious findings by health professionals and studies of African women and women from other ethnic groups, but reported on findings for the included ethnic groups separately were also included.

### Exclusion criteria

Excluded articles consisted of those that included African women and women from other ethnic groups within the overall sample, but did not report on findings for the included ethnic groups separately. Studies that reported research carried out in Western countries, only reported differences in time to presentation/diagnosis by race and did not explore factors accounting for these differences, only described interventions in relation to increasing uptake of cancer screening and/or improving early presentation/detection rates were also excluded. African women’s perceptions of cancer without information on early presentation and diagnosis were excluded.

### Data extraction and quality appraisal

Data were extracted systematically from all eligible papers through the use of standardized Data Extraction Forms (DEFs) developed by a research team [21]. Two standardized forms were used for qualitative and quantitative studies that merited inclusion in this review. Data were extracted independently by CPA, DB and DYQ and verified by other authors. Any disparities in prevalence data were resolved by consensus-based discussions among the authors.

The quality of the quantitative studies was assess based on National Institute of Health (NIH) Quality Assessment Tool for Observational Cohort and Cross-Sectional Studies [22]. Qualitative studies were appraised using different quality criteria [23]. Both extraction forms assessed the aims of the study, sampling procedure, data collection methods, analysis approach and limitations. The qualitative extraction form further assessed the level of critical self-reflection about biases and the extent to which findings from the study could be transferred to other settings or groups. The quantitative form appraised the reliability, validity and generalizability of the quantitative papers.

### Synthesis of findings

Findings from qualitative and quantitative articles were integrated into themes. Thematic synthesis of the qualitative studies [24] provides explicit and clear links between conclusions and the text of primary studies used in a review by ensuring rigor discussion of article content. This enabled analytical abstraction of results. Findings from the quantitative papers were absorbed within the themes using the multi-source synthesis method [25]. Multi-Source Synthesis is an analytical technique that enhances transparency when synthesizing quantitative and/or contextual data, thus providing a platform for comparison between studies [25]. It also serves as a systematic guide in synthesizing data from their primary studies to give a meaningful and broad understanding of the subject.

## Results

### Study characteristics

**Fig 1:**
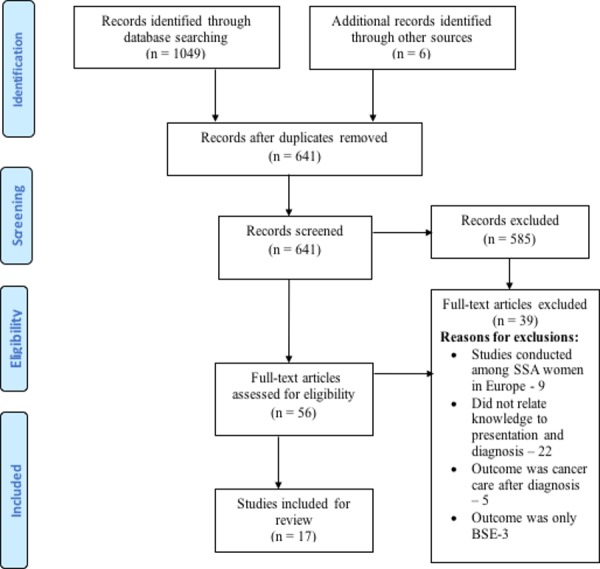
Flow chart for citations reviewed during different phases of the systematic review.

The flow of information through the various stages of the review is represented in Fig 1. Seventeen (17) articles merited inclusion for this review. Out of these, 2 [26, 27] employed qualitative methods, one (1) mixed methods [42], whiles fourteen (14) were quantitative studies [28–34, 35, 36–41] and were published from 2002 to 2015, Fig 2.

**Fig 2:**
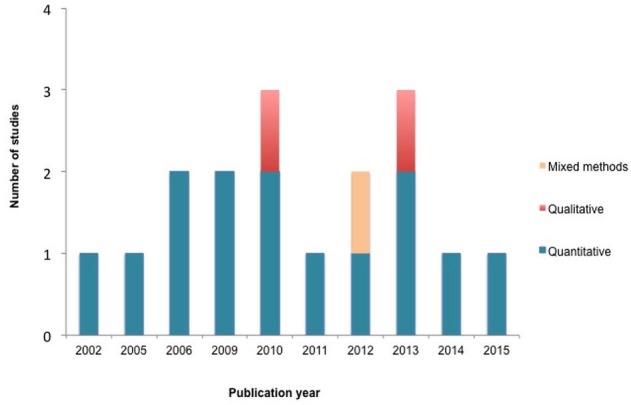
Type of included studies per publication year.

Nine (9) of the quantitative articles were descriptive cross-sectional surveys [29, 32, 33, 35–38, 40, 42] whereas 4 articles employed correlation methods [28, 30, 31, 34]. The two remaining papers [39,41] were quantitative surveys. One of the qualitative articles involved the use of focus group discussion (FGDs) [26] to explore the knowledge and attitudes of BC and its early detection measures whiles the other used in-depth interviews. Details on methodology, sample size, sampling and analysis are provided in Tables 1 and 2.

**Table 1:** Details of qualitative study included in the review.

Authors, country of study	Methods	Participants/ Setting	Findings	Quality
**Muthoni & Miller (2010) [26], Kenya**	Focus group Interview, framework analysis	Females rural & urban, Kenyans (20-60years)	***Low level of awareness and knowledge***	Good
			• ↓ level of awareness in BC • ↓ knowledge about warning signs • ↓ knowledge in causes • ↓ knowledge in CSE & SBE • ↓ knowledge about treatment	
	Sampling: Convenience/purposive			
	Size: 6–7 in 4 groups			
		
			***Misconception about BC***	
			• Wrong perception of early detection benefits	
**Mbuka-Ongona & Tumbo (2013) [27], Botswana**	In-depth interviews	Breast cancer patients	***Low level of awareness and knowledge***	Good
	Sampling: Purposive	(>18years), Urban	• ↓ knowledge of BC prior to diagnosis	
	Size: 12			
			***Misconception and misinformation about breast cancer*** • Fear of diagnosis and death	
			***Other barriers to late presentation***	
			• Health practitioners lack of seriousness about breast cancer • Influence of traditional healers in delaying treatment seeking • Infrequent screening activities • Difficulty in assessing healthcare–disadvantaged by living in remote areas	

**Table 2:** Details of quantitative and mixed method studies included in the review.

Authors	Methods	Participants	Findings	Quality
**Ademola et al (2010) [35], Nigeria**	Cross-sectional, interview administered questionnaire, SPSS Sampling: Convenient, Purposive; Size: 1194	Females Nigerians, Rural (20-45years)	***Low level of awareness and knowledge*** • ↓awareness in knowledge about BC, 39.2% • ↓awareness in BC screening, 46.5% • ↓knowledge about causes and symptoms of BC, 8.0% and 1.5% • ↓knowledge about screening procedure, 0.5% ***Misconceptions about causes and treatment*** • Believe can be transmitted sexually, 49.2% • Perceive BC affects only Caucasians, 55.3% • Believe BC has no cure, 33.9%	Fair
**Akhigbe & Omuemu (2009) [33], Nigeria**	Cross-sectional survey, interview administered questionnaire, x^2^ analysis; Sampling: Random; Size: 393	Females doctors, nurses, pharmacists, lab scientists, radiologist, Nigerians (>20years)	***Low level of awareness and knowledge*** • Not aware BC is common worldwide, 36.4% • Poor knowledge in risk factors, 55.0% • No knowledge in mammography as screening method, 67.3% • ↓knowledge of SBE as screening method, 45.8% • Did not know mammography as diagnostic method, 19.3% ***Low practice/ participation in BC screening*** • Never had mammogram, 96.9% • Never performed SBE, 33% ***Influence of age on BSE*** • BSE ↑ with age: 65.2% among 20–29 to 100% among those from 60years and above	Good
**Bologun & Owoaje (2005) [29], Nigeria**	Cross-sectional survey, interview administered questionnaire, SPSS, x^2^ analysis Sampling: Purposive; Size: 281	Females traders Nigerians (16–80years)	***Low level of awareness and knowledge*** • Not aware of BC, 68.3% ***Low practice/ participation in BC screening*** • Most did not practice SBE, 81.9% ***Influence of age and education on BSE*** • ↑Awareness of SBE among 50-59years: 38.7% compared to those <30 and >60 Association between education and knowledge in SBE: p = 0.045	Fair
**Clegg-Lamptey et al (2009) [31], Ghana**	Survey, interview administered questionnaire; Sampling: Convenience purposive; Size: 101	Females Ghanaians (20-84years)	***Low level of awareness and knowledge*** • No knowledge in BC, 57.6% Misconceptions about causes and treatment Only 29.4% sought medical consultation ***Reasons for delay presentation*** • Fear of mastectomy 34.8%, financial incapability 28.8%, death 25.8%, ignorance 28%	Fair
**Maree & Wright (2010) [34], South Africa**	Correlational survey. Self-administered questionnaire, Pearson x^2,^ Spearman’s rho; Sampling: Convenience purposive; Size: 565	Females, South African women (>18years)	***Low level of awareness and knowledge*** • Poor knowledge in BC, 44.8%; • ↓ knowledge of sign/symptom of BC, 20.1%; ↓ knowledge of warning signs, 47.7% ***Low practice/ participation in BC screening*** • 57.8% examined their breast monthly ***Misconceptions about causes and treatment*** • 12.6% associate lump in the breast as cancer ***Poor health seeking behaviour*** • 61.3% have to be encourage to seek healthcare • 20.7% needed permission to seek healthcare • 78.8% would wait less than 1week to tell of any suspicion of BC ***Lack of information about BC;*** Only 6.7% healthcare professionals providing information about cancer	Fair
**Okobia et al (2006) [32], Nigeria**	Cross-sectional survey, interview administered; Sampling: Random; Size: 1000	Females semi-urban community (15-91years)	***Low level of awareness and knowledge*** • Low overall knowledge score, 42.3% • ↓ knowledge of presence of BC as painless lump, 21.4% • ↓ knowledge SBE as screening method, 43.2% • Small portion indicated Mammography as screening method ***Low practice/ participation in BC screening;*** SBE, 34.9%; mammography, 0%; CBE, 9.1% ***Misconceptions about causes and treatment;*** BC attributed to evil spirit, 40.0%; infection, 2.9% ***Poor health seeking behaviour*** • 8.2% would visit alternative health practitioners for care of BC **Knowledge significantly related to educational level**	Good
**Oluwatsin & Oladepo (2006) [30], Nigeria**	Survey, interview administered questionnaire Sampling: Multistage random; Size 420	Females, rural setting (20–60years)	***Low level of awareness and knowledge*** • 48% not aware that cause of BC is unknown • ↓ of warning sign of BC, 73.7% ○Only 1.9% identified painless lump as early warning sign ○Only 1.5% identified pain as early warning sign • ↓ knowledge of any treatment of BC, 90.7% unaware • ↓ knowledge of benefit of early treatment, 41% • ↓ knowledge of screening/early detection measures: SBE 6.4%; CBE 1.2%; mammography 0% ***Low practice/ participation in BC screening; only 18*.*9 practices***	Fair
**Pillay (2002) [28], South Africa**	Survey, interview administered, x^2^ multivariate & 3-way analysis of variance Sampling: Random Size 140	Females, rural and urban setting (21-59years)	***Low level of awareness and knowledge*** • ↓ knowledge of screening/early detection measures: SBE 50%; CBE >50% • Unaware Doctor can test for BC: 1/3^rd^ ***Influence of type of residence and education on practice and knowledge*** • BSE practice in Rural: 31.4%, in Urban 82.9% p<0.00 • Prefer a traditional healer; Rural: 37.7%, Urban: 10.1% p<0.001 • Less than grade 8 education had significantly less knowledge on BC	Fair
**Ibrahim& Oludara (2011) [37], Nigeria**	Cross-sectional Sampling: All patients studied; Size: 201	Breast cancer patients, Urban; (23-104years)	***Delay > 3 months before initial medical consultation*: *81*.*6%*** ***Influence of socio-demographic characteristics on late presentation*** • Not being married (OR = 2.054, 95% CI: 0.252–16.759) • Primary level of education (OR = 3.059, 95% CI: 0.962–9.731) • Being pre-menopausal (OR = 1.861, 95% CI: 0.383–9.039)	Good
**Suh et al (2012) [38], Cameroon**	Cross-sectional, standardized questionnaire; Sampling: Convenient and consecutive; Size: 120	Adults > = 20years, Semi-Urban	***Low level of awareness and knowledge*** • ↓ knowledge of causes of BC • ↓ knowledge level of BSE as BC prevention method, 36.7% • ↓ knowledge of risk factor for BC, 45%	Good
**Azubuike & Okwuokei (2013) [36], Nigeria**	Cross-sectional; Sampling: Purposive, stratified and simple; Size: 365	Women in reproductive age (19-49years), Urban	***Low level of awareness and knowledge*** • ↓ knowledge of any risk factor, 26%; Only 49.7% knew up to 3 risk factors • Only 15% identified 6–7 signs and symptoms; 22.5% had no knowledge of any sign or symptom • ↓ Low knowledge of importance of breast detection strategies; 3.4% strongly agreed • ↓ Low knowledge level of secondary preventive measures; 43.3% could not identify a single preventive measure ***Low practice/ participation in BC screening;*** 53.4% practiced none • ↑ knowledge level increased tendency to practice any of the early preventive procedures (P < 0.01)	Fair
**Maree et al (2013) [39] South Africa**	Quantitative survey; Sampling: Convenient; Size: 299	Women presenting at CC and BC prevention &screening project, (> = 18 years), Rural	***Low level of awareness and knowledge*** • Lack of awareness of the signs of breast cancer ***Low practice/ participation in BC screening*** • ↓ BSE and only 5% practiced monthly ***Age and literacy associated screening practices***	Fair
**Morse et al (2014) [41], Tanzania**	Quantitative survey; Sampling: Convenient; Size: 299	Outpatients at government-supported hospitals (18-55years), Urban	***Low level of awareness and knowledge*** • 44% never heard of BSE • 32% never heard of CBE ***Misconceptions about causes and treatment*** • Belief of not being at risk of BC • Beliefs in common myths and folklore regarding the causes of breast cancer ***Low practice/ participation in BC screening*** • 40.5% of those heard never practiced BSE • 60.4% heard but never received CBE	Good
**Pace et al (2015) [40], Rwanda**	Cross-sectional; Sampling: All participants; Size: 365	159 (144 involved in analysis)	***Median total delay = 15 months*** ***Influence of education and alternative treatment on late presentation*** • Low educational level (OR, 4.88; 95% CI, 1.72–13.88) Seeing a traditional healer before a medical officer or nurse (OR, 4.26; 95% CI, 1.56–11.60)	Good
**Opoku, Benwell and Yarney (2012), [42], Ghana**	Cross-sectional; Cluster and systematic sampling; Size: 500; Quantitative -474	General population (40-70years), Urban	***Low level of awareness and knowledge*** • ↓ Knowledge Level of BC and BC screening • ↓knowledge of signs and symptoms of the disease ***Misconception about causes and risk factors of BC*** ***Low practice/ participation in BC screening;*** BSE 32%, CBE 12%; mammography 2%	Good

### Study participants

A total of 6681participants were recruited and involved in the14 included quantitative studies. The least number of participants in a study was 110 women [31] and the largest consisted of 1194 participants [35]. One qualitative study [26] involved 6–7 participants in 4 focus group discussions. However, the total number of participants involved was not reported whereas the other qualitative study recruited 12 participants for in-depth interviews. A total of 500 subjects participated in the mixed method study [42]. Participants were recruited from both urban and rural settings with varied background characteristics. The ages of participants ranged from 18 to 91years.

### Barriers to early presentation and diagnosis of breast cancer

Several barriers to early presentation and diagnosis of BC were reported across all studies. Table 3 shows the summary of barriers to late presentation of BC reported by included studies whiles Table 4 shows respondents’ personal reasons for late presentation or diagnosis of BC. Majority of the articles revealed limited knowledge of symptoms and risk factors of BC among study subjects. According to Gates, Lackey and Brown [43], a limited knowledge of breast cancer signs and symptoms are generally associated with delayed presentation. The results of our included studies indicate a strong association between knowledge of BC with delayed presentation among women with breast cancer. Ademola et al [35] reported 39.2% of participants to have had knowledge in BC whereas Akhigbe and Omuemy [33], showed that, even though 45.8% participants had knowledge of breast self-examination (BSE), majority (55.0%) of participants had poor knowledge on the risk factors of BC. Balogun and Owoaje [29] reported a majority (68.3%) of subjects who were not aware of BC. Other barriers reported were limited knowledge of screening practices (BSE, clinical breast examination [CBE], and mammography) and varied misconceptions, misinformation and lack of information. Some of the misconceptions include associating lump in the breast as cancer [28], BC attributed to evil spirit and infection [32], beliefs in folklore and myths regarding the causes of BC [41], fear of death upon diagnosis [27], belief that BC can be transmitted sexually, perception that BC affects only Caucasians, and the notion that BC has no cure [35]. Factors such as limited screening facilities in communities and poor health attitudes were also reported [27], as barriers to late presentation. Pace et al [40] presented results of both individual and health system barriers that results in late diagnosis and or treatment options for BC. Poor health seeking behavior as well as varied socio-demographic characteristics was not left out across studies as significant barriers to BC diagnosis and treatment.

**Table 3:** Summary of barriers late presentation and diagnosis of BC.

Barriers	Number of studies (n = 17)	Percentage
***Awareness and knowledge***		
• Limited knowledge of BC	13	76.4
• Limited knowledge of screening practices (BSE, CBE, mammography)	5	29.4
• Misconceptions, misinformation, lack of information	8	47.1
***Low participation in BS screening and examination practices***	8	47.1
***Facility and health service related***		
• Limited screening facilities in community	1	5.8
• Poor health attitude	1	5.8
***Poor health seeking behavior***	3	17.6
***Socio-demographic characteristics***		
• Age	2	11.8
• Education	4	23.5
• Marital status	1	5.8
• Residence	1	5.8
• Pre-menopausal	1	5.8
***Socio-cultural***		
• Fear of cancer diagnosis, death, stigma, diagnosis procedure	5	29.4
• Belief in other sources of treatment	4	23.5
• Gender roles and household decision making	1	5.8

**Table 4:** Personal reasons for late presentation/ diagnosis.

Reasons	Pace et al [40]	Opoku, Benwell &Yarney [42]	Azubuike &Okwuokei [36]	Ibrahim & Oludara [37]	Okobia et al [32]	Clegg-Lamptey et al [31]	Muthoni& Miller [26]
Not bothered to check	x						
Ignorance of nature of disease			x	x	x	x	
Financial constraints	x	x		x		x	x
Belief in alternative treatment	x			x			
Busy/forgot	x		x				
Fear of having cancer/death	x	x	x			x	x
Fear of stigma	x						
Unaware of appropriate facilities / procedures	x	x	x		x		
Health Inaccessibility							x
Fear of being examined (unwilling to expose body)	x	x	x				
Not referred		x					
Fear of procedure		x		x		x	
Attitude of health staff		x					
Culture interferences							x

### Awareness and knowledge of breast cancer

Majority of the studies (15) reported lower participants’ knowledge of the causes, symptoms, early detection and treatment of BC [26, 28–37, 39, 40–42]. Studies that recruited health care professionals [33] revealed that, 55% of subjects had knowledge of BC risk factors, 67% had good understanding of the burden of BC and 73% correctly identified BC as the most common cancer in women [33]. About 66% of subjects also revealed that BC was more severe than other forms of cancers in the study by Oluwatosin and Oladepo [30].

Awareness of BSE and CBE were reportedly very low in some studies [26,29–31,35,42,41]. Majority of the participants involved in all included studies had little or no knowledge of mammography and BSE [30–32,35,42,41,39]. A study, however, reported 81% of its participants to have had some knowledge of mammography as a diagnostic procedure rather than screening or early detection measure whereas 24% lacked details such as what mammography is and how it is done, the benefits, appropriate age to start mammography screening and its benefits to women [33]. Knowledge on the benefits of early detection measures was also low among participants [26, 28,30].

Some studies showed that participants with higher educational attainment were more knowledgeable about BC issues than those with lower levels of education and or had no formal education [32,33]. Women in urban areas were more informed of BC than their colleagues in rural settings [28,30,35]. Higher levels of education were also significantly associated with BSE among respondents [29,32].

### Sources of breast cancer information

The sources of respondent’s information on BC were investigated in some studies [30,32,34,42,41], Table 5. In the studies conducted by Oluwatosin and Oladepo [30] as well as Okobia and colleagues [32], the major (31% vs. 5.4%) source of the respondents BC knowledge was the television whereas in [34], most of the respondents indicated primary healthcare as their chef source of information. BC information leaflets and physicians were the main sources of BC information in Okobia [32]. In contrast, studies conducted in Ghana and Tanzania [41,42] indicated radio (39.8% vs. 36.4%) as the main source of BC knowledge among respondents. In [30], women obtained BC information from community elders, neighbors and friends (15.4%) and from people who have had the disease in the past (5.2%).

**Table 5:** Source of Breast Cancer Information.

Source	OKobia et al, 2006 [32]	Oluwatosin&Oladepo, 2006 [24]	Maree & Wright 2010 [34]	Opoku, Benwell & Yarney, 2012 [42]	Morse [41]
Healthcare professionals	21.1%	4.4%	6.75	13.9%* /12.4%**	7.1%
Primary healthcare	-	-	30.7%	-	-
Cancer awareness group	6.0%	-	3.9%	-	-
Feminist organisation group	6.7%	-	-	-	-
Supervisors of breast cancer	-	5.2%	-	4.7%	-
Television	31.0%	5.4%	13.9%	-	
Radio	-	-	20.6%	20.5%	36.4%
Leaflets/newspaper	27.1%	-	-	39.8%	0.9%/1.8%
Elders/Friends/Neighbours	-	15.4%	-	5.1%	8.9%
Church /Religious organisation	8.1%	-	-	6.5%	- -
Family members	-	-	-	7.8%	-

*Nurses and midwives

**doctors

### Perceptions of breast cancer

Varied perceptions of BC were also reported across studies. A large number of participants perceived BC as very serious form of cancer [30,34] or deadly [35]. In the study by [30], 36% of study subjects were reported to have indicated that ‘*it kills fast’*. In some studies, participants were reported to have the view that, African women were not susceptible to BC. About 55% believed it affected Caucasians only [26,35]. Other studies reported that, respondents were of the view that BC is caused by infection, evil spirit and could be transmitted sexually [32,35]. Results from qualitative studies, were not different from those of quantitative design. Studies reported that, participants were not convinced treatment could save women from losing their breasts or death [28,30,32]. In one study, a participant noted that *“We normally know that if someone gets cancer they will end up dying”* [30]. A young lady posited that; *“My understanding of cancer is that it is like a blue gum tree*, *you cut it*, *a new growth begins to sprout from the trunk*, *so when the breast is removed*, *new cancer begins again and spreads to other parts of the body”*. *“In fact*, *AIDS is much better than breast cancer*,*”* another asserted, *“because at least you can manage it with drugs*, *you don’t develop maggots like cancer*. *So I say anything that has no medicine is not good*.*”* [26]. In some studies, participants were reported to have believed that mastectomy was the only treatment for BC [31] whereas participants in others studies revealed that BC was nor curable [30,32,35].

### Health seeking behaviour

The review revealed poor health seeking behaviors among women in SSA and this influence the early presentation and diagnosis of breast cancer. Poor health seeking behaviors were as a result of socio-cultural factors, fear of being diagnosed cancer and death from cancer, as well as traditional practices, Table 3.

#### Sociocultural factors and traditions

A study, [26], revealed that low self-esteem and the awkward feeling of knowing one has a BC and its associated stigma causes trauma and thus deters most women from seeking healthcare when needed. Some women were reported to have indicated that they prefer their health been a secondary issue than their families been stigmatized because of their BC status. The responsibilities of women in African culture make health a low priority and prevent them from seeking healthcare and/or attending educational forums. In one study, 78.8% of women would wait less than one week to tell of any suspicion of BC, and 20.7% needed permission from spouses in seeking healthcare [34].

#### Fear

Fear was reported as a contributing factor for women’s failure to seek treatment for BC [32–34,35,37,39,42]. This included fear of cancer diagnosis, fear of being examined by a physician and fear of being stigmatized if diagnosed positive. Participants indicated that the fear of mastectomy and death resulting from been diagnosed of BC prevented them from consulting their physicians or reporting at a health center [31,32].

#### Preference for alternative treatment

The fear, stigma and cost involved in diagnosis and treatment in Africa compels women to seek for alternative source of BC care including traditional healer and or herbalist. Pace et al., [40] reported that, participants prefer to see a traditional healer before a medical officer and that subjects’ level of education and preference for alternative treatment was closely associated. According to Pillay, [28] 37.7% of rural and 10.1% of urban participants prefer a traditional healer for breast cancer diagnosis and treatment (p<0.001).

Okobia et al, [32] also reported that 8.2% of participants consult alternative health practitioners for treatment. The influence and dominance of traditional healers in rural African communities does not only act as a barrier to orthodox treatment but also results in delay to seek treatment [27].

#### Socio-demographic characteristics and access to healthcare

Socio-demographic characteristics such as age, marital status, women education and type of residence was reported as impediments to BC treatment. Age as a barrier to late presentation was described by [29,33,39]. Maree et al [39] outlined the extent to which age and literacy were associated with screening practices. Bologun and Owoaje, [29], revealed that, awareness of SBE among 50-59years was 38.7% compared to those between the ages of <30 and >60. Akhigbe and Omuemu [33] reported that BSE practice increases with age of participants; 65.2% among 20–29 to 100% among those from 60years and above. Unmarried women (OR = 2.05, 95% CI: 0.25–16.76) were shown to have higher odds of late presentation than their married counterparts [37]. Pace et al, [40] explicitly indicated that, low education level (OR, 4.88; 95% CI, 1.72–13.88) increases women’s odds of late presentation whereas less than grade 8 of education was also shown to have a significant association with less knowledge of BC [28,32]. Level of education was also reported to influence BSE practices among women [29].

Barriers owing to inadequate screening programs, attitude of health personnel’s and access to healthcare were also intimated to contribute to late presentation of BC. Difficulty in accessing healthcare as a result of living in a remote area and the lack of seriousness of health professionals about BC was shown to be a barrier to early presentation [27]. Maree and Wright [34] indicated that 78.8% of women would wait less than 1week to tell of any suspicion of BC, and 20.7% needed permission from spouses in seeking healthcare further delays presentation.

### Quality of papers reviewed

Authors rated majority of the studies to be of good or high quality based on our assessment criteria [26,27,32,33,36–38,40–42]. These studies described in detail the design and methodology used, the process of recruiting participants, study setting, clear and detailed presentation of findings, study limitations, were unlikely to affect the reliability and validity of study findings. Studies that were rated lower quality [26,28–31, 34,35] were papers without explicit research questions, limited information on data analysis, failure to describe subject recruitment processes, small sample and lack of justification of sample size and other issues that could lead to a high risk of bias and the generalizability of the study.

## Discussion

This review sought to identify health seeking behaviors and other contributing factors to delays in BC detection among African women. We identified knowledge gap as an important contributing factor for late presentation and early detection measures. Knowledge is an important determinant of healthcare utilization and this association has been previously established. Studies also reveal that this barrier to BC treatment and management is not only evident among African women but also in other developed countries including UK, USA, Canada, Hong Kong and India where an association between poor knowledge and health literacy with late detection of BC and case presentations persist [44–48].

In some African countries discrepancies exist between the knowledge of participants in urban and rural settings. Whereas urban middle-income women identified lifestyle factors such as stress, sedentary behaviour, and dietary factors as possible risk factors for BC, rural women indicated, remnants of milk retained in the breast, keeping money or a mobile telephone inside their braziers, witchcraft, evil spirits and punishment from the gods as factors contributing to BC [26,35]. The possibility of urban dwellers attaining higher education than their rural counterparts could account for the differences in knowledge between among African women. Again, women in the urban areas could have more credible and useful health information about diseases including BC than those in the rural areas. Two studies [32–34] found higher level of education to be positively associated with high knowledge in BC among participants. This is consistent with previous evidence that showed a positive association between higher education and increased knowledge about health [49–51].

There is evidence from this review that women in Africa have a low level of knowledge on early detection measures for BC, which appears to affect their practices and involvement. The combined effect of poor knowledge of early detection measures and low health literacy has been identified as influencing the disease in some developed countries [15,33,48]. A review undertaken in San Francisco [52] on delay versus help seeking for BC symptoms found that women do not always realize the significance of BC symptoms. Women participants in a cohort study in London [53], which sought to determine factors that influence delayed presentation of BC, revealed that even though participants identified significant symptoms of BC they had to be persuaded by others to see a doctor. This suggests that, despite women appreciating the seriousness and burden of BC [30,34,35], influencing factors are key in getting needed help. Again, women would need to be encouraged before seeking BC health despite the consequences of the outcome of treatment and its potential financial constraints on their families.

What is of much concern in sub Saharan Africa is the availability of accurate health information. Sources of information determines how accurate the information is, and these information shapes their perception and beliefs about breast cancer, its causes and the need to participate in screening and other important preventive practices. A study undertaken among urban residents in Chicago [48], reported that misconceptions about breast lumps and decreased access to health care were associated with prolonged delay in seeking health care. Similarly, beliefs and perceptions about cancers among patients attending radiotherapy in Delhi, India [46], revealed that majority of the patients believed that cancer is caused by God’s curse (59%), evil eye (60%) and past or present sins (37%). About 27.4% of the patients indicated that cancer is infectious and about 57% of them revealed close contact with infected cancer patient were the cause of their illness. These findings are consistent with the result of this review. Perception of African women has a positive correlation with their health seeking behaviors [26, 30–32,35]. Studies also reveal that black women living in Brent and Harrow in UK hold on to such superstition [15] concerning BC.

Studies reported that, most women received BC information mainly from television (31%), clinics (31%) and health professionals (21%) [32,34]. Other sources of BC information were from elders, friends and neighbors who serve as a source of support in seeking advice, knowledge and encouragement about health issues. One study reported elders, friends and neighbors as the main source of BC information among Nigerian rural women in Ibadan [30]. Other studies conducted in Nigeria revealed television sources as the main platform for assessing BC information among respondents in a semi urban community [32]. This disparity might be attributed to the different socioeconomic status within these two communities. Women from semi urban communities are more likely to afford television whereas those in the rural areas may rely on opinion leaders such as to elders in the community, friends and neighbors for the same information. This phenomenon results in late acquisition of BC information and late presentation at the facility since women are likely to do so when symptoms are presented. The more knowledgeable an opinion member on risk factors and causes of BC, the more likely for them to influence audience to see a physician. In parts of Africa where community elders revere herbal and traditional medicine than orthodox treatment, it is doubtful whether these elders, friends and neighbors will assist BC women to seek appropriate health care rather than directing them to herbal, religious places or other approaches over conventional medicine. Source of information and it accuracy impacted women’s attitude toward screening involvement and presentation.

This prompt the need for educational campaigns among African women to enhance their knowledge of BC and benefits of early detection and treatment. The Breast Health Global Initiative (BHGI) [54] strives to develop evidence based, economically feasible and culturally appropriate guidelines that can be used in nations with limited health care resources to improve BC outcomes. These guidelines could be adapted to develop BC education programs in African countries to realize the goal of increasing its awareness by making use of the limited resources available.

Decision to seek medical attention was found to be a barrier to early BC diagnosis and presentation in this review. This is shaped by the women’s preferences for alternative treatment. A study reported that only 29% indicated their preparedness to seek medical attention upon identifying such signs and symptoms whereas others prefer traditional treatment (46%) for BC care [28,31,32]. Women in rural settings are even more likely to seek help from traditional healers due to deficit in healthcare facilities in rural areas. Previous evidence suggests that women who seek alternative treatment mostly present late with severe clinical stages and sometimes with 8 to 10 months duration of symptoms [55,25].

Sociocultural factors such as influence of husbands and partners were also important in determining early detection and treatment. The influence of societal factors on access to healthcare is also mediated through their opportunities to education, income, occupation, control over earnings and participation in decision-making. Gender norms and values of women in society determine their status within the household and the community at large, and influence their access to healthcare [56]. In the traditional African society, women had to seek permission from their spouses before seeking treatments at the hospital and attending health education forums [34]. Generally, women relegate their health to second with regards to family responsibilities [52,53,57]. This poses an important barrier to the early detection and diagnosis of breast cancer. Women in these situations will find difficulty to practice BSE and to present early the health facility when symptoms are detected.

Access difficulties, long distances and residing in remote areas pose difficulties for women with respect to access to BC diagnosis and treatment. Living in rural areas is associated with greater distances and high cost of transport to access healthcare services [58,59]. In some cases, women who are screened for BC fail to honor referrals secondary care for mammography. Previous evidence from SSA studies, identified transport cost, availability of transport and travel time as strong correlates of health care utilization among women [60,58,61]. In SSA, where a chunk of the population resides in remote areas, with difficult access to health facilities, accessing BC programmes organized at the facilities becomes a challenge. Remote communities are also hard to reach with screening programmes due to transportation challenges.

The strength of this review reflects the various themes identified and their consistency with studies from other developed countries on the subject area [15,44,45,53,62,63]. Our inability to include all studies conducted in Africa might affect the generalization of our findings. Generalization about African women’s health seeking behavior with respect to BC could be problematic since diverse cultures and tribes with different characteristics exist among African countries [64]. However, we believe that the results identified are compelling and shows consistent pattern of BC knowledge deficit among African women.

## Conclusion

Generally, knowledge inadequacy of BC and its early detection measures continue to be one of the most important factors in determining women’s attitude towards BC screening and treatment. Understanding the benefits of early detection and presentation of BC among women was poor across all studies. Sources of information, knowledge of early detection measures, sociocultural beliefs and traditions revealed women’s perceptions of BC. Societal traditions and beliefs play an important role in women’s BC perceptions, screening measures and treatment. The findings of this study prompt educational campaigns among African women to enhance their knowledge of BC, benefits of early detection and treatment.

## Supporting information

S1 Appendix Search strategy used to generate included studies. (DOCX)
