# Supplementary material for: Barriers to early presentation and diagnosis of breast cancer among African women living in sub-Saharan Africa
Source: PLoS One. 2017 Feb 13;12(2):e0171024. doi: 10.1371/journal.pone.0171024 (PMC5305236; doi:10.1371/journal.pone.0171024)
Supplement: S1 Appendix — (DOCX) [file pone.0171024.s001.docx]

**Appendix1: Search Strategy to generate included papers**

|  | **AND** | **AND** | **AND** |
| --- | --- | --- | --- |
| Neoplasms (SH)  neoplasm* (free text)  cancer* (free text)  tumor* (free text) | African Continental Ancestry Group (SH)  black* not (Blackwell) (free text),  minority group* (free text)  ethnic minorit* (free text)  “Black Minority Ethnic” (free text)  “BME” (free text) | Perception (SH)  Social Perception (SH  perception* (free text)  social perception* (free text)  opinion* (free text)  Attitude to Health (SH)  attitude* (free text)  social value* (free text)  social norm* (free text)  Culture (SH)  belief* (free text)  understanding* (free text)  language* (free text)  communicat* (free text)  fear* (free text)  mistrust (free text)  trust (free text)  cultur* (free text)  relig* (free text)  knowledge* (free text)  barrier* (free text)  embarrass* (free text)  fatalism (free text)  fatalistic (free text)  income (free text)  socioeconomic* (free text)  depriv* (free text)  educat* (free text)  poor* (free text)  poverty (free text) | Early Diagnosis (SH)  Early Detection of Cancer (SH)  “Late presentation” (free text)  “Early presentation” (free text)  “Early diagnos*” (free text)  “Late diagnos*” (free text)  “early detection cancer”  delay* (free text) |
